# Cardiometabolic Risk-Driven Diagnosis of Cerebellar Infarction Presenting as Isolated Transient Vertigo: A Case-Based Review

**DOI:** 10.3390/reports9040320

**Published:** 2026-09-22

**Authors:** Pavol Skáčik, Vladimír Nosáľ, Štefan Sivák, Egon Kurča

**Affiliations:** Department of Neurology, University Hospital Martin, Jessenius Faculty of Medicine in Martin, Comenius University Bratislava, 036 01 Martin, Slovakia

**Keywords:** cardiometabolic risk factors, transient vascular vertigo, posterior circulation stroke, carotid artery stenosis, obesity, type 2 diabetes mellitus

## Abstract

**Background and Clinical Significance**: Vertigo is a common manifestation of posterior circulation ischaemia, and obesity, type 2 diabetes mellitus, hypertension, and dyslipidaemia are established cardiometabolic risk factors for the underlying atherosclerotic disease. Brief, isolated episodes may be diagnostically challenging because bedside findings can be normal after symptom resolution, despite clinically significant cerebrovascular disease. We present a case in which an adverse cardiometabolic risk profile—rather than the clinical presentation itself—prompted the vascular imaging that established the diagnosis, together with a targeted review of comparable reports; **Case Presentation**: We investigate the case of a 67-year-old man with type 2 diabetes mellitus, hypertension, dyslipidaemia, obesity, and known asymptomatic right internal carotid artery (ICA) stenosis presented after a single spontaneous episode of rotatory vertigo lasting five minutes, which resolved completely before assessment. Neurological and structured neuro-otological examinations were normal. Because of his cardiometabolic and vascular risk burden, further imaging was pursued: CT angiography demonstrated progression of the right ICA stenosis to 85%, chronic occlusion of the left vertebral artery, and mild stenosis of the right vertebral artery, and brain MRI subsequently confirmed a small acute ischaemic lesion in the left cerebellar hemisphere. Management combined carotid artery stenting with a personalised, multidisciplinary secondary prevention plan addressing his full cardiometabolic risk profile. At three months, he remained asymptomatic, with NIHSS and modified Rankin Scale scores of 0. A targeted, CABARET-guided literature review of six comparable case reports showed that our patient carried the most extensive cardiometabolic burden yet had the mildest and most transient presentation, in contrast to the recurrent or progressive symptoms typical of the comparator reports; **Conclusions**: A single brief episode of isolated vertigo may be the only manifestation of acute posterior circulation ischaemia, even when bedside findings are normal. In patients with an adverse cardiometabolic and vascular risk profile, a reassuring examination should not preclude further neurovascular investigation, and early, risk-guided recognition—supported by personalised, multidisciplinary care—may provide an important opportunity for stroke prevention.

## 1. Introduction and Clinical Significance

Obesity, type 2 diabetes mellitus, arterial hypertension, and dyslipidaemia are established cardiometabolic risk factors for accelerated atherosclerosis and may allow cerebrovascular disease to progress silently [1]. Vertigo and dizziness are common clinical manifestations of posterior circulation stroke, including infarction or transient ischaemia within the vertebrobasilar territory. Diagnostic recognition is particularly challenging in the absence of accompanying focal neurological signs, creating a potentially misleading discrepancy between an apparently reassuring clinical examination and clinically significant underlying cerebrovascular pathology [2]. Formal diagnostic criteria for vascular vertigo and dizziness, distinguishing acute prolonged from transient presentations, have been proposed by the Bárány Society [3].

Mechanistically, this link is direct rather than coincidental. Cardiometabolic-driven atherosclerosis frequently affects multiple arterial beds concurrently, and when both anterior and posterior circulation are involved, the resulting dyscirculation can compromise perfusion of the vertebrobasilar territory supplying the brainstem and cerebellum. This region houses a disproportionately dense concentration of structures responsible for balance—the vestibular nuclei, cerebellar vestibular pathways, and their connections—supplied by end-arteries with limited collateral reserve. Even a modest or transient reduction in flow can therefore disrupt these structures selectively, making vertigo one of the most common, and often earliest, symptoms of vertebrobasilar hypoperfusion or infarction, regardless of how small the ischaemic insult is.

Transient isolated vestibular symptoms may precede posterior circulation stroke, often in patients with an adverse cardiometabolic risk profile, and the diagnostic utility of the Head Impulse–Nystagmus–Test of Skew (HINTS) battery is substantially reduced in this transient population compared with its established performance in acute prolonged vestibular syndrome [4]. Neuroimaging may compound rather than resolve this discrepancy: magnetic resonance imaging (MRI), particularly diffusion-weighted imaging, may yield false-negative findings during the acute phase, especially within the first 48 h, and small strokes presenting with severe vertigo remain associated with a substantial rate of false-negative MRI findings even beyond this early window [5,6].

Vascular and cardiometabolic comorbidities should independently raise suspicion of a central aetiology even when the bedside examination and initial imaging are unremarkable [7]. Many patients encountered in everyday clinical practice carry a comparably adverse cardiometabolic risk profile—often with minimal, atypical, or no referable symptoms at all—and are appropriately managed with routine ambulatory risk-factor surveillance rather than emergency-level vascular imaging. What distinguished the present patient was not the risk profile in isolation, but the occurrence of a new, spontaneous, transient vestibular event—however brief and fully resolved by the time of assessment—in a patient already known to have atherosclerotic cerebrovascular disease. A transient neurological symptom of this kind carries the same short-term stroke-risk implications as a transient ischaemic attack, irrespective of how minimal or non-specific it appears at the bedside, and this risk is compounded rather than offset by an adverse cardiometabolic profile. It was this combination—a new transient vestibular symptom superimposed on a high-risk vascular substrate—rather than the cardiometabolic risk profile alone, that justified urgent rather than ambulatory investigation, and that ultimately established the diagnosis of an acute posterior circulation stroke. We report this case, together with a targeted Case-Based Review Standards (CABARET)-guided literature review, to illustrate how the combination of transient vestibular symptoms and cardiometabolic risk should prompt urgent, rather than elective, vascular assessment.

Clinical Significance: This case highlights that, in patients with obesity, type 2 diabetes mellitus, hypertension, and dyslipidaemia, even a minimal and fully transient vestibular symptom should be regarded as a potential marker of clinically significant, multi-territorial atherosclerotic cerebrovascular disease and assessed with the same urgency as a transient ischaemic attack, rather than deferred to ambulatory work-up. In this high-risk population, recognising the combination of a transient vascular vertigo presentation and cardiometabolic risk-factor burden—rather than relying on symptom severity or bedside findings alone—as the trigger for prompt vascular imaging may allow earlier stroke prevention.

## 2. Case Presentation

A 67-year-old man with type 2 diabetes mellitus, obesity, arterial hypertension, and dyslipidaemia presented to the emergency department after a single spontaneous episode of rotatory vertigo (2023), without an identifiable provoking factor, lasting approximately five minutes. The symptoms resolved completely before arrival. He reported no accompanying otological, cranial nerve, or focal neurological symptoms, and no preceding head trauma, infection, or similar previous episodes.

His relevant vascular and metabolic history included a known asymptomatic, haemodynamically insignificant stenosis of the right internal carotid artery (ICA), first identified on carotid imaging in 2021, together with diabetic peripheral polyneuropathy of the lower limbs, evidenced by reduced deep tendon reflexes and impaired vibration sense.

General and neurological examinations were unremarkable, with no focal neurological deficit. A structured bedside neuro-otological assessment showed no central pattern: there was no spontaneous nystagmus, the head impulse test was negative bilaterally, and no skew deviation was present. Oculomotor examination, including smooth pursuit and saccades, was normal. The Dix–Hallpike and Pagnini–McClure tests were negative for positional vertigo, and Romberg testing showed only mild postural instability.

At presentation, blood pressure was 160/90 mmHg and heart rate was 64 beats per minute. Oxygen saturation was normal, and he was afebrile. Electrocardiography showed sinus rhythm without ischaemic changes, and routine biochemistry, serum electrolytes, full blood count, and inflammatory markers were within normal limits. During hospitalisation, his fasting lipid profile confirmed marked dyslipidaemia, with total cholesterol 8.55 mmol/L, triglycerides 5.26 mmol/L, HDL cholesterol 1.13 mmol/L, and LDL cholesterol 5.17 mmol/L; homocysteine was mildly elevated at 15.63 µmol/L. His glycaemic profile demonstrated poor metabolic control, with capillary glucose values of 8.4 mmol/L in the morning, 13.5 mmol/L at midday, and 16.2 mmol/L in the evening.

Despite these reassuring bedside and laboratory findings, further imaging was pursued because of his cardiometabolic risk profile and known carotid disease, rather than on the basis of the clinical presentation alone. Non-contrast CT of the brain showed clinically silent ischaemic changes in the territory of the right ICA, without any previously recognised neurological event (Figure 1A).

CT angiography of the intra- and extracranial vessels demonstrated marked progression of the right ICA stenosis compared with the 2021 study, from a haemodynamically insignificant lesion to an 85% stenosis; the degree of stenosis was confirmed by digital subtraction angiography (DSA), and revascularisation was indicated (Figure 1B).

The same study also showed an approximately 50% stenosis of the right vertebral artery, subsequently estimated at approximately 30% on DSA, together with chronic occlusion of the left vertebral artery (Figure 1C).

The coexistence of anterior- and posterior-circulation vascular disease—including severe right ICA stenosis, chronic occlusion of the left vertebral artery, and non-flow-limiting stenosis of the right vertebral artery—suggested impaired collateral circulation and a possible haemodynamic mechanism for transient vertebrobasilar hypoperfusion.

Given this vascular substrate, the patient was admitted for further investigation and empirical secondary prevention, comprising antiplatelet therapy with acetylsalicylic acid 100 mg once daily together with a temporary course of therapeutic-dose low-molecular-weight heparin.

Brain MRI performed during admission, including fluid-attenuated inversion recovery, diffusion-weighted imaging, and apparent diffusion coefficient sequences, confirmed the suspected posterior-circulation ischaemic lesion: it demonstrated a small focus of restricted diffusion in the left cerebellar hemisphere, consistent with an acute ischaemic lesion and temporally related to the episode of vertigo (Figure 2).

Following digital subtraction angiography, the severe right ICA stenosis was treated with carotid artery stenting. The approximately 30% stenosis of the right vertebral artery was managed conservatively, while the chronic left vertebral artery occlusion required no endovascular intervention.

Alongside this endovascular treatment, a secondary prevention strategy was designed around the patient’s individual cardiometabolic risk profile, and included optimisation of antiplatelet therapy, blood pressure control to a target below 140/90 mmHg, and lipid-lowering treatment with rosuvastatin. Hyperhomocysteinaemia was treated with vitamins B6 and B12 and folic acid, and cardiac screening was performed to exclude a potential embolic source.

Lifestyle measures—addressing the modifiable components of his cardiometabolic risk—included dietary modification, weight reduction, and at least 150 min of moderate-intensity aerobic activity per week. At the three-month follow-up, the patient remained free of recurrent vertigo or balance disturbance and was clinically stable with comprehensive vascular risk-factor management. He had no focal neurological deficit, with a National Institutes of Health Stroke Scale score of 0 and a modified Rankin Scale score of 0.

### 2.1. Literature Search Strategy

This report was structured as a case-based review, following the Case-Based Review Standards (CABARET) recommendations [8]. A PubMed/MEDLINE search, conducted on 25 July 2025 with no restriction on publication date, used the terms ‘isolated transient vertigo’ and ‘transient vertebrobasilar vertigo’ and yielded 249 records. Titles and abstracts were screened to identify case reports and case series describing vertigo potentially related to vertebrobasilar arterial disease; approximately ten underwent full-text assessment. Reports were included if they provided patient-level data, demonstrated or adequately supported posterior circulation ischaemia, and reported sufficient detail on episode pattern, ocular motor findings, neuroimaging, vascular/cardiometabolic risk profile, treatment, and outcome for comparison with the present case; reports were excluded if the full text was unavailable or posterior circulation ischaemia was not sufficiently supported. Six English-language articles met these criteria and were included (Supplementary Table S1); reference lists of selected reports were also searched manually for additional publications.

This was a non-systematic, single-reviewer, single-database search, without independent dual screening, formal quality appraisal of included reports, or restriction to a defined publication period; English-language restriction may have excluded relevant non-English reports. Given the small number and heterogeneity of included cases, findings are presented as a narrative comparison rather than a pooled analysis, and should be regarded as hypothesis-generating.

### 2.2. Results of Review

Luan et al. reported up to 20 attacks. The reported patients were aged 43–67 years, with no clear sex predominance. In contrast to our patient, who experienced a single brief episode without recurrence, all comparator cases involved recurrent or progressive symptoms. Halmagyi’s patient had two brief episodes before a severe brainstem stroke [9]. Luan et al. reported up to 20 attacks per day over four days [10]. Kerber et al. reported more than 200 stereotyped attacks over approximately two years [11]. Other patients experienced recurrent vertigo over several weeks or months [12,13], Kim et al. described four transient episodes before a prolonged attack [14].Thus, the present case was distinct in that a single, fully resolved episode was associated with an acute ischaemic lesion.

Vascular risk profiles varied. Our patient had multiple established risk factors, including type 2 diabetes mellitus, hypertension, dyslipidaemia, and obesity. Similar atherosclerotic risk profiles were reported by Luan et al. and Lee et al. [10,13]. Kerber et al.’s patient had hypertension and Parkinson disease [11], whereas Halmagyi’s patient had no recognised vascular risk factors [9]. In Kim et al.’s case, paroxysmal atrial fibrillation was identified as the probable embolic source [14]. Among the reported cases, our patient carried the most extensive cardiometabolic burden—the co-occurrence of type 2 diabetes mellitus, hypertension, dyslipidaemia, and obesity—supporting cardiometabolic risk-factor clustering as a plausible substrate for the multi-territorial atherosclerotic disease observed on angiography.

Detailed acute ocular motor findings were available in only four reports. Halmagyi’s patient had no nystagmus and negative head impulse and Dix–Hallpike tests during an earlier transient episode, although a later stroke produced total horizontal gaze palsy [9]. Luan et al. likewise reported an initially normal examination, with an NIHSS score of 0, no nystagmus, and negative head impulse and Dix–Hallpike tests [10]. Kerber et al.’s patient had normal ocular motor findings between attacks, while skew deviation and diplopia occurred only during symptomatic episodes [11]. By contrast, Lee et al. and Kim et al. documented abnormal findings, including spontaneous nystagmus and positive head impulse testing [13,14]. Gomez et al. did not provide patient-level ocular motor data [12]. These observations show that transient vertebrobasilar ischaemia may occur despite an initially reassuring bedside examination.

Large-vessel pathology in the comparator reports predominantly involved the posterior circulation. Reported abnormalities included vertebral artery occlusion, severe vertebral or basilar stenosis, contralateral vertebral hypoplasia, and AICA occlusion [9,10,11,12,14]. Kim et al. reported normal intracranial angiography, with a presumed cardioembolic mechanism [14]. In contrast, our patient had concurrent anterior- and posterior-circulation disease, comprising severe right ICA stenosis, chronic left vertebral artery occlusion, and mild right vertebral artery stenosis, consistent with his broader cardiometabolic risk burden. This combination was not reported in the comparator cases.

MRI findings varied according to the vascular mechanism. Halmagyi et al. reported bilateral medial medullary, pontine, and cerebellar infarction [9], Luan et al. described posterior cerebral and cerebellar infarcts [10], and Lee et al. reported bilateral pontine infarction [13]. Kerber et al.’s patient had no MRI-visible infarct despite recurrent symptoms, supporting a transient haemodynamic mechanism without permanent tissue injury [11]. Kim et al. reported small hippocampal and basal ganglia infarcts in the setting of presumed embolism [14]. In the present case, MRI demonstrated a small acute infarct in the left cerebellar hemisphere.

Treatment reflected the presumed mechanism. Medical therapy included antiplatelet agents, anticoagulation, and intensive vascular and cardiometabolic risk-factor management [9,12,13,14]. Endovascular treatment was used in patients with severe symptomatic vertebrobasilar stenosis after medical therapy proved insufficient [10,11]. Our patient underwent carotid artery stenting, while the posterior-circulation lesions were managed conservatively.

Outcomes were generally favourable, although reporting was mostly descriptive. Luan et al. documented an mRS score of 0 at six months [10], while Kerber et al. reported complete resolution of recurrent attacks after stenting [11]. Other patients improved gradually or retained minor neurological deficits [9,13,14]. In the series by Gomez et al., two of six patients developed brainstem infarction, one of which was fatal [12]. Our patient remained asymptomatic and neurologically intact at three months, with NIHSS and mRS scores of 0.

Overall, the reviewed reports show that episode duration, frequency, and an apparently benign clinical presentation do not reliably exclude cerebrovascular disease, and that an adverse cardiometabolic risk profile, as in our patient, may be among the most reliable indicators for pursuing vascular imaging regardless of symptom severity.

## 3. Discussion

This case demonstrates that a single, brief episode of isolated vertigo may correspond to acute cerebellar ischaemia despite a normal neurological and ocular motor examination. Its principal relevance lies not in the episode’s duration, but in the discrepancy between an apparently benign presentation and the underlying cerebrovascular pathology—and in the role of cardiometabolic risk in resolving that discrepancy.

The main contribution of this case is therefore not the cerebellar infarction itself, but the demonstration that, in a transient vestibular syndrome, an adverse cardiometabolic and vascular risk profile can be the decisive factor that establishes a structural ischaemic diagnosis—not merely a coincidental comorbidity. Here, this risk profile was the primary trigger for vascular and parenchymal imaging in a patient whose brief, fully resolved symptom and normal bedside findings would otherwise have appeared reassuring. This reframes vascular risk-factor burden as an active determinant of diagnostic and management decisions in acute vestibular syndromes generally, given the possibility of a vascular cause even when symptoms are transient.

Transient vestibular symptoms may represent an early or incomplete manifestation of posterior circulation ischaemia, and may precede stroke or accompany significant vertebrobasilar disease [9,10,11,12,13,14,15]. As symptom frequency and progression vary widely, neither a single episode nor the absence of recurrence is sufficient evidence of a benign cause.

The present case also illustrates the limitations of bedside examination in transient presentations. Tests used to distinguish peripheral from central acute vestibular syndromes are most informative while symptoms and ocular motor abnormalities are present. When vertigo has resolved before examination, the findings may be entirely normal. HINTS-based assessment should therefore not be used in isolation to exclude vascular disease in an asymptomatic patient with a brief, resolved episode.

In this setting, vascular and cardiometabolic risk assessment becomes particularly important. Our patient’s risk profile—type 2 diabetes mellitus, arterial hypertension, dyslipidaemia, and obesity, together with known atherosclerotic carotid disease—reflects the clustering of cardiometabolic risk factors increasingly recognised as a driver of silent, multi-territorial atherosclerotic progression [1]. In such patients, a single, fully resolved episode and an entirely normal bedside examination may, taken in isolation, appear reassuring; it was specifically this cardiometabolic burden, rather than the clinical presentation itself, that justified further investigation despite the absence of focal neurological signs, and that ultimately led to the diagnosis of an acute posterior circulation stroke. The ABCD2 score and similar clinical tools may support risk estimation, but they cannot replace clinical judgement or direct assessment of the cerebral and cervical vasculature [3,7]. The threshold for vascular imaging should remain low when transient spontaneous vertigo occurs in a patient with a substantial cardiometabolic and vascular risk burden.

This recommendation cannot, however, reasonably be extended to universal advanced vascular imaging for every patient with an elevated cardiometabolic risk profile, given the scale of this population and the finite imaging and angiography resources of most healthcare systems. At a population level, a more feasible strategy is two-fold: first, structured education of neurologists and primary care physicians on the possibility of transient vascular vertigo in high-risk patients, supported by validated risk-stratification tools such as the ABCD2 score, to raise the index of suspicion and guide selective rather than universal referral for vascular imaging; and second, thorough clinical risk assessment with careful exclusion of alternative neurological and non-neurological causes of vertigo before advanced imaging is pursued, so that imaging resources are directed toward patients in whom the combination of symptom pattern and vascular risk burden makes a central cause genuinely plausible. In the present case, the decision to pursue detailed vascular and parenchymal imaging was also shaped by the local care pathway: the patient was assessed and managed by a neurologist with specific expertise in vestibular and balance disorders and acute vertigo, which likely lowered the practical threshold for pursuing a complex vascular work-up compared with a general emergency or primary care setting. This reflects a referral- and expertise-dependent element of management that limits the direct generalisability of our approach and should be considered when interpreting its broader applicability.

Parenchymal and vascular imaging provide different, complementary information. MRI may identify acute tissue injury, whereas CT angiography, MR angiography, or catheter angiography may reveal the vascular substrate responsible for transient or recurrent symptoms. A normal early MRI does not exclude posterior circulation stroke, particularly when lesions are small or imaging is performed soon after symptom onset [6,16]. Conversely, clinically important arterial stenosis or occlusion may be present without a visible infarct. Repeat MRI and dedicated vascular imaging should therefore be considered when clinical suspicion persists.

The vascular findings in our patient were complex. The acute lesion was located in the left cerebellar hemisphere, while angiography demonstrated severe right ICA stenosis, chronic left vertebral artery occlusion, and mild right vertebral artery stenosis. This combination suggests widespread atherosclerotic disease and potentially reduced collateral reserve, consistent with the extent of his underlying cardiometabolic risk burden, although the precise mechanism of the cerebellar infarct cannot be established with certainty. The carotid stenosis should therefore be interpreted as an important coexisting vascular lesion rather than assumed to be the direct cause of the vestibular presentation.

Management should be guided by the presumed mechanism and the vascular lesion identified. Acute treatment may include antiplatelet therapy, anticoagulation for a confirmed cardioembolic source, intravenous thrombolysis, or thrombectomy in eligible patients. Endovascular treatment of intracranial vertebrobasilar stenosis is generally reserved for selected patients, as intensive medical therapy remains the preferred initial approach in most cases [17]. In the present patient, posterior circulation disease was managed conservatively, while the severe right ICA stenosis was treated with carotid artery stenting. The role of parenteral anticoagulation in the acute phase of ischaemic stroke remains limited and should be reserved for selected indications rather than routine use as seen in the presented case [18]. The concurrent use of antiplatelet therapy, short-term anticoagulation, and high-intensity statin treatment in a patient with type 2 diabetes mellitus and diabetic polyneuropathy also illustrates the need to anticipate drug interactions and cumulative bleeding or metabolic risk when combining cardiovascular and metabolic therapies, reinforcing the value of a multidisciplinary approach involving neurology, cardiology, vascular medicine, and diabetology.

Irrespective of the immediate mechanism, secondary prevention is essential. Blood pressure control, lipid lowering, diabetes management, smoking cessation where applicable, weight reduction, physical activity, and appropriate antithrombotic treatment reduce the risk of subsequent vascular events [19]. A personalised, multidisciplinary secondary prevention plan, tailored to the patient’s full cardiometabolic risk profile rather than to the presenting neurological symptom alone, is likely to be particularly relevant in this population.

This case supports a practical clinical message: transient spontaneous vertigo should not be assessed solely according to its duration, recurrence, or apparent severity. In patients with relevant vascular risk factors, a normal examination after symptom resolution does not reliably exclude ischaemia [20]. Careful risk assessment and appropriately selected neurovascular imaging may reveal clinically important disease that would otherwise remain unrecognised, and this principle may be especially relevant for the broader population of patients with obesity, diabetes, hypertension, and dyslipidaemia, in whom cardiometabolic risk factor burden—rather than symptom severity alone—should guide the threshold for vascular imaging.

Several limitations should be acknowledged. This report is based on a single case with a relatively short (three-month) follow-up, which limits generalisability and does not allow assessment of longer-term outcomes. The decision to pursue comprehensive vascular imaging was influenced by referral to a vestibular-specialist neurologist, introducing a referral- and expertise-dependent selection element that may not be replicable in all clinical settings. The accompanying literature comparison was a targeted, non-systematic review of a small number of heterogeneous published cases, and comparisons across reports were further limited by inconsistent documentation of ocular motor findings and standardised outcome measures. These limitations should be considered when extrapolating the clinical message of this case to broader practice.

This report is limited by its single-case design, short follow-up. Comparison across reports was further restricted by inconsistent documentation of ocular motor findings and clinical outcomes. Prospective studies using standardised bedside assessment, neuroimaging protocols, and outcome measures are needed to improve risk stratification in patients with transient vertigo.

## 4. Conclusions

A single, fully transient episode of isolated vertigo may be the only manifestation of acute posterior circulation ischaemia, even when bedside findings are normal. In patients with relevant vascular and cardiometabolic risk factors, a reassuring examination should not preclude further neurovascular investigation. Early recognition, guided by a personalised assessment of the patient’s overall cardiometabolic and vascular risk burden, may provide an important opportunity for stroke prevention, and underscores the value of multidisciplinary care in patients with obesity and cardiometabolic disease.

Further work should focus on developing and validating structured risk-stratification pathways—combining clinical risk scores such as ABCD2 with cardiometabolic risk burden—to identify which patients with transient, minimal vestibular symptoms warrant urgent vascular imaging, so that resources are directed efficiently rather than applied universally. Prospective, multicentre studies with standardised bedside and neuroimaging protocols and longer-term outcome follow-up are needed to determine the real-world diagnostic yield and cost-effectiveness of such a risk-guided approach, and to clarify its generalisability beyond specialist vestibular referral settings.

## Figures and Tables

**Figure 1:**
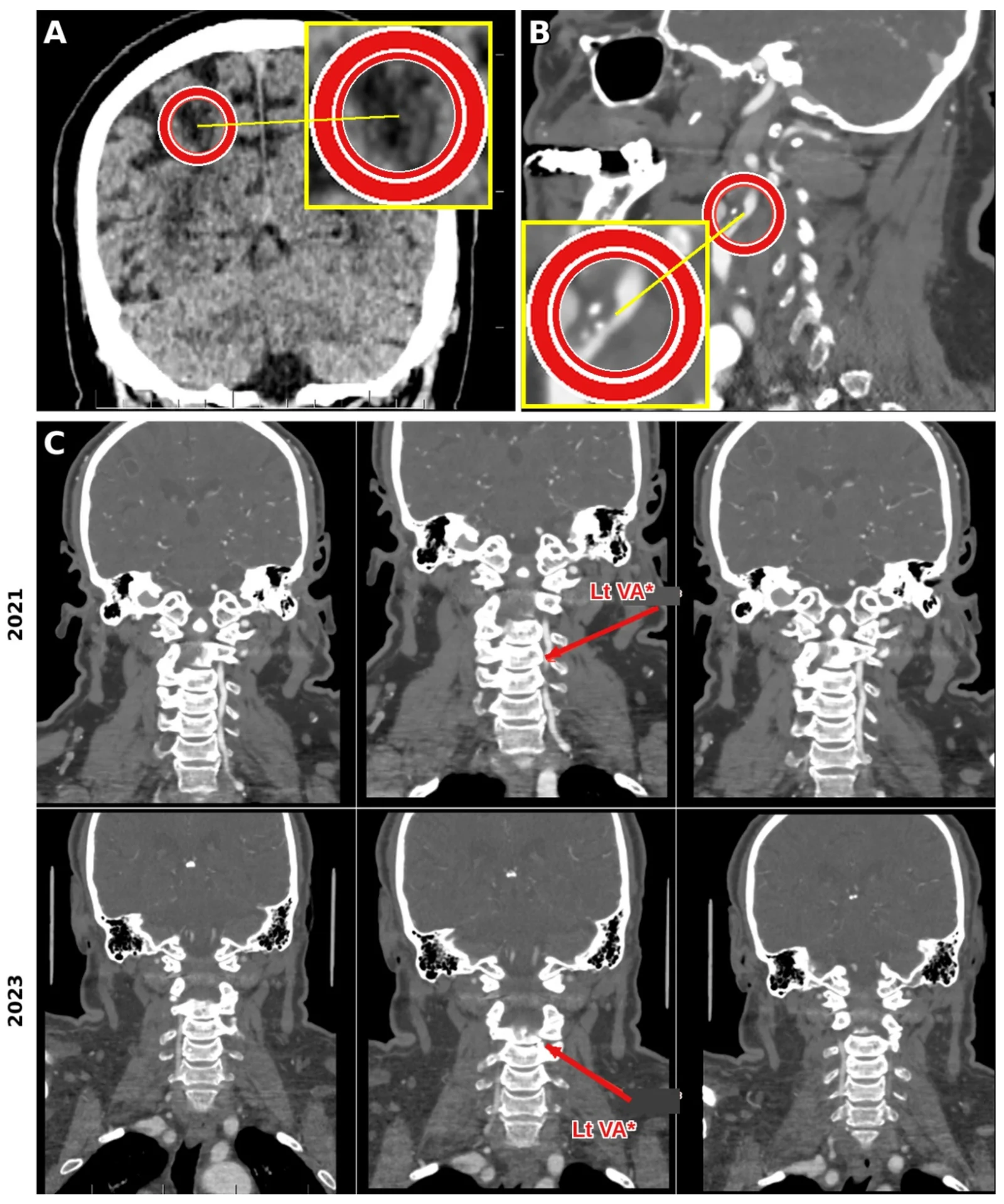
Neuroimaging findings. (**A**) Coronal non-contrast CT of the brain showing a clinically silent post-ischaemic lacune in the right internal carotid artery territory (red circle). (**B**) Sagittal CT angiographic reformation demonstrating high-grade stenosis of the right internal carotid artery (red circle). (**C**) Coronal CT angiographic images obtained in 2021 and 2023 showing interval occlusion of the left vertebral artery (Lt VA*). The occlusion was considered chronic at the time of the 2023 examination following review by an interventional neuroradiologist. ICA, internal carotid artery; Lt, left; VA, vertebral artery. Lt VA* indicates the same anatomical location of the left vertebral artery, which was patent in 2021 and occluded in 2023.

**Figure 2:**
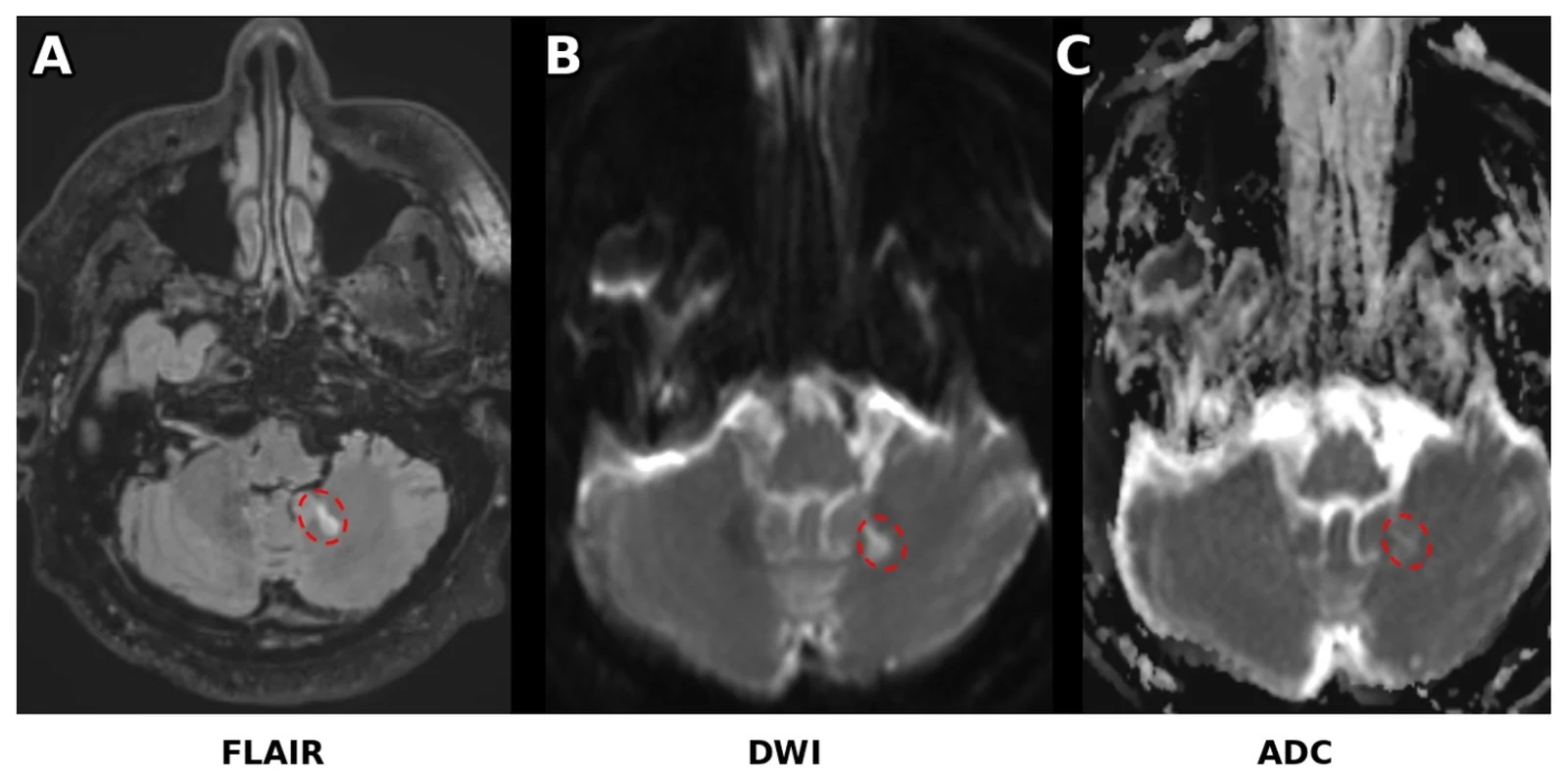
Brain MRI demonstrating acute ischaemia. Axial MRI of the posterior fossa: (**A**) fluid-attenuated inversion recovery (FLAIR), (**B**) diffusion-weighted imaging (DWI), and (**C**) apparent diffusion coefficient (ADC) map. A small lesion in the left cerebellar hemisphere shows restricted diffusion, with DWI hyperintensity and corresponding ADC hypointensity (red circles), consistent with acute ischaemia and temporally related to the episode of vertigo.

## Data Availability

The data supporting the findings of this study are available from the corresponding author upon reasonable request. Data are not publicly available due to privacy and ethical restrictions.

## Supplementary Materials

The following supporting information can be downloaded at https://www.mdpi.com/article/10.3390/reports9040320/s1.

## Abbreviations

The following abbreviations are used in this manuscript:

ICA	Internal carotid artery
VA	Vertebral artery
DSA	Digital subtraction angiography
CTA	Computed tomography angiography
MRI	Magnetic resonance imaging
DWI	Diffusion-weighted imaging
ADC	Apparent diffusion coefficient
FLAIR	Fluid-attenuated inversion recovery
HINTS	Head Impulse–Nystagmus–Test of Skew
ASA	Acetylsalicylic acid
LMWH	Low-molecular-weight heparin
NIHSS	National Institutes of Health Stroke Scale
mRS	Modified Rankin Scale
CABARET	Case-Based Review Standards
