# Supplementary material for: Cardiometabolic Risk-Driven Diagnosis of Cerebellar Infarction Presenting as Isolated Transient Vertigo: A Case-Based Review
Source: Reports (MDPI). 2026 Sep 22;9(4):320. doi: 10.3390/reports9040320 (PMC13692451; doi:10.3390/reports9040320)
Supplement: Supplementary file 1 [file reports-09-00320-s001.zip › reports-4563603-supplementary.pdf]

Supplementary Table S1. Expanded comparison of the present case with published case reports/series

Demographics, episode pattern, vascular/cardiometabolic risk profile, acute clinical/ocular motor findings, anterior- and posterior-circulation imaging, MRI localisation, treatment, and outcome in the present case and comparable published case reports/series identified via a CABARET-guided PubMed/MEDLINE search. This table reproduces Table 1 of the main text at expanded size for legibility; the content remains unchanged.

Source [ref]	Age/Sex	Episodes (number, duration, progression)	Vascular/cardiometabolic risk profile	Acute clinical/oculomotor findings	Anterior-circulation imaging	Posterior-circulation imaging	MRI brain localisation	Treatment	Outcome
Present case	67 y, M	Single episode, 5 min, complete resolution, no progression	Type 2 diabetes mellitus, obesity, hypertension, dyslipidaemia	Nystagmus: absent. HIT: negative bilaterally. Skew: negative. Normal pursuit/saccades; mild Romberg titubation	Right ICA 85% (progressed from haemodynamically insignificant)	Chronic left VA occlusion; non-flow-limiting right VA stenosis	Acute lesion, vestibular pathways (left cerebellar hemisphere)	Continuation of ASA + transient LMWH → carotid artery stenting + secondary prevention	No neurological deficit; asymptomatic at 3-month follow-up (NIHSS 0, mRS 0)
Halmagyi [9]	Adult, M	2 preceding brief attacks (minutes; ~1 h) → 3rd event = acute stroke	None	Preceding attack: no reported nystagmus, HIT negative, Dix–Hallpike negative (skew not tested). Acute event: total horizontal gaze palsy; vertical movements, convergence, pupils intact	None reported	Left VA occlusion at lower pons → thrombus extension to proximal basilar artery	Bilateral medial medulla, dorsal mid-pons, cerebellum	Aspirin → deterioration at 24 h (dysarthria, dysphagia, hemiparesis) → heparin infusion; thrombectomy attempted, abandoned; anticoagulation 2 weeks	No formal mRS/NIHSS; at 1.5 years, hemiplegia "almost resolved," persistent horizontal gaze paresis
Luan et al. [10]	67 y, M	Up to 20 episodes/day over 4 days, non-positional	Hypertension, untreated dyslipidaemia (statin-intolerant)	NIHSS 0; no nystagmus (post-meclizine); Dix–Hallpike negative; HIT negative; no dysmetria/ataxia	None reported	Left VA (V4) 80–90% stenosis; hypoplastic right VA (<1 mm)	Right PCA and superior cerebellar artery territories (occipital, temporal, cerebellar)	Dual antiplatelet therapy (refractory) → urgent intracranial balloon angioplasty + drug-eluting stent	mRS 0 at 6 months; no recurrence
Kerber et al. [11]	63 y, M	>200 stereotyped attacks over ~2 years; vertigo/tinnitus then skew diplopia ~30–40 s later; each attack 1–30 min, several/day	Parkinson disease, hypertension	Resting tremor, mild bradykinesia, cogwheel rigidity (PD). Nystagmus: not reported. Head-thrust: normal, symmetric VOR. Skew/diplopia present during attacks. Normal oculomotor function and hearing; tandem-gait difficulty, no dysmetria	Right VA occluded 5 mm proximal to vertebrobasilar junction (atherosclerotic); left VA tortuous, no significant atherosclerosis	Proximal basilar artery stenosis 70%→90%, immediately proximal to AICA origin	MRI brain normal (no fixed lesion despite recurrent attacks)	Prior ASA + anticoagulation (failed) → balloon-mounted intracranial stent (3.5×8 mm) of basilar stenosis	Attacks abolished; no further episodes at 3-month follow-up; mRS/NIHSS not reported

**Source [ref]**	**Age/Sex**	**Episodes (number, duration, progression)**	**Vascular/cardiometabolic risk profile**	**Acute clinical/oculomotor findings**	**Anterior-circulation imaging**	**Posterior-circulation imaging**	**MRI brain localisation**	**Treatment**	**Outcome**
Gomez et al. [12]	5 M, 1 F	Recurrent isolated vertigo ≥4 weeks	Not specified in detail	Not systematically reported (series predates the formal HINTS description, 2009)	None reported	Focal basilar stenosis or diffuse vertebrobasilar slow flow (catheter-confirmed in 3)	Not systematically reported	Warfarin (n=5), aspirin (n=1)	2/6 brainstem infarction (1 fatal); mRS/NIHSS not reported
Lee et al. [13]	43 y, F	Monthly episodes (~20 min) over 12 months → acute prolonged attack	Smoking, poorly controlled diabetes, hypertension	Direction-fixed right-beating nystagmus (fixation-enhanced); HIT abnormal for posterior canals only (bilateral, worse left), normal for anterior canals; skew negative	None reported	Left VA occlusion + right AICA occlusion; mid-basilar stenosis	Bilateral acute pontine infarcts (vestibular nuclei/fascicles)	Aspirin + aggressive vascular risk-factor management	Gradual symptom improvement; mRS/NIHSS not reported
Kim HA et al. [14]	67 y, F	4 episodes (minutes) over 10 days → prolonged 3-day attack	Paroxysmal atrial fibrillation (newly identified)	Spontaneous right-beating horizontal-torsional nystagmus (upbeat component); HIT positive (left); no dysmetria, ophthalmoparesis, or weakness	Normal MRA	Normal MRA (including AICA)	Left hippocampus + basal ganglia (atypical, embolic pattern)	Anticoagulation (for atrial fibrillation)	Resolved over several days of hospitalisation; mRS/NIHSS not reported

HIT, head-impulse test; ICA, internal carotid artery; VA, vertebral artery; AICA, anterior inferior cerebellar artery; PCA, posterior cerebral artery; CTA, CT angiography; MRA, MR angiography; ASA, acetylsalicylic acid; LMWH, low-molecular-weight heparin; mRS, modified Rankin Scale; NIHSS, National Institutes of Health Stroke Scale; PD, Parkinson disease; VOR, vestibulo-ocular reflex. "Not specified" indicates the finding was not reported in the available source, not that it was necessarily normal or absent. Gomez et al. (1996) predates the formal description of the HINTS examination (2009) and could not, by definition, report individual head-impulse, nystagmus, or skew findings.
